# Stable episodic memory and higher education do not influence the rate of Alzheimer's disease pathology as measured by plasma p‐tau217

**DOI:** 10.1002/alz.71920

**Published:** 2026-11-04

**Authors:** Bárbara Avelar‐Pereira, Nicola Spotorno, Anna Orduña Dolado, Divya Bali, Annelie Nordin Adolfsson, Niklas Mattsson‐Carlgren, Sebastian Palmqvist, Shorena Janelidze, Oskar Hansson, Lars Nyberg

**Affiliations:** ^1^ Aging Research Center Karolinska Institutet and Stockholm University Stockholm Sweden; ^2^ Department of Psychiatry and Behavioral Sciences School of Medicine Stanford University Stanford California USA; ^3^ Clinical Memory Research Unit Department of Clinical Sciences Malmö Lund University Lund Sweden; ^4^ Department of Clinical Sciences, Psychiatry Umeå University Umeå Sweden; ^5^ Memory Clinic Skåne University Hospital Malmö Sweden; ^6^ Wallenberg Center for Molecular Medicine Lund University Lund Sweden; ^7^ Umeå Center for Functional Brain Imaging Umeå University Umeå Sweden; ^8^ Department of Medical and Translational Biology Umeå University Umeå Sweden; ^9^ Department of Diagnostics and Intervention Umeå University Umeå Sweden

**Keywords:** blood‐based biomarkers, education, episodic memory, plasma p‐tau217, preclinical Alzheimer's disease

## Abstract

**INTRODUCTION:**

Blood‐based biomarkers, particularly phosphorylated tau 217 (p‐tau217), can detect early Alzheimer's disease (AD) neuropathological changes. Recent work has shown that p‐tau217 is lower in individuals with stable cognition and higher education. These factors may protect against AD pathology.

**METHODS:**

We evaluated whether individuals with stable episodic memory and higher education exhibited lower levels of or slower increases in p‐tau217 using longitudinal data from the population‐based Betula study (*n* = 1005; 1531 samples).

**RESULTS:**

P‐tau217 increased with age over 10 years (*p* < 0.001), with the steepest increase seen in individuals with accelerated episodic‐memory decline (*p* < 0.001). There were no differences in p‐tau217 trajectories between individuals with lower or higher education (*p* = 0.386) or with well‐maintained or age‐typical decline in episodic memory (*p* = 0.885). The null finding for education was replicated in the BioFINDER‐2 cohort (*p* = 0.14).

**DISCUSSION:**

Plasma p‐tau217 can detect early pathological changes in population‐based settings, but our findings do not support the notion that higher education or well‐maintained memory protects against AD neuropathological changes.

## Highlights

- Plasma p‐tau217 increased longitudinally over 10 years in a population‐based sample.
- Individuals with accelerated episodic memory decline show the steepest p‐tau217 increase.
- Educational attainment does not moderate longitudinal p‐tau217 trajectories.
- Well‐maintained episodic memory is not associated with reduced p‐tau217 accumulation.
- Null findings for education were replicated in the independent BioFINDER‐2 cohort.

## 1 BACKGROUND

Alzheimer's disease (AD) is a neurodegenerative disorder characterized by the accumulation of extracellular amyloid beta (Aβ) plaques and intracellular aggregates of hyperphosphorylated tau.^1^ Over time, Aβ and tau pathology disrupt synaptic function and lead to neuronal loss, resulting in cognitive impairment, notably episodic‐memory decline.^2^, ^3^ AD is characterized by a long preclinical phase, and there is growing interest in blood‐based biomarkers that can provide diagnostic information.^4^ Tau phosphorylated at threonine 217 (p‐tau217) has been found to closely correlate with brain Aβ burden, discriminate AD from other neurodegenerative disorders with high accuracy, and predict cognitive decline in non‐demented samples.^5^, ^6^, ^7^, ^8^, ^9^, ^10^, ^11^, ^12^, ^13^ Still, existing longitudinal studies have primarily examined p‐tau217 trajectories in relation to amyloid and cognitive status within clinic‐based cohorts. Comparatively little is known about whether individual differences in long‐term cognitive trajectories, or established resilience factors such as education, are associated with the level or rate of p‐tau217 accumulation, particularly in cognitively heterogeneous, population‐based samples.

A recent large‐scale study^14^ used 11,486 blood samples to investigate p‐tau217 burden in relation to age and several other variables including cognition and education. P‐tau217 concentration levels indicative of AD neuropathology were (1) higher in older age groups, (2) higher for individuals with mild cognitive impairment (MCI) than for cognitively unimpaired individuals, and (3) highest for older individuals with only primary education. Thus, individuals with well‐maintained episodic memory and higher educational attainment may display lower levels and slower accumulation of p‐tau217, possibly reflecting reduced AD pathology. Here we test these predictions using longitudinal data from the population‐based Betula study in northern Sweden.^15^, ^16^

First, baseline differences and 10‐year longitudinal changes in plasma p‐tau217 were examined in relation to three previously defined episodic‐memory aging profiles over 15 years^17^: (1) age‐typical memory decline (“average”); (2) accelerated memory decline (“decliners”); and (3) high‐stable memory performance (“maintainers”). Based on findings that decliners have elevated risk of AD and that maintainers have reduced risk,^18^ we hypothesized that decliners would exhibit the greatest increase in p‐tau217 over time and maintainers the lowest. Second, in view of recent cross‐sectional findings suggesting that higher educational attainment might be associated with lower increases in p‐tau217,^14^ we explored whether educational attainment modified age‐related longitudinal increases in plasma p‐tau217.

**RESEARCH IN CONTEXT**

1. **Systematic review**: A recent large‐scale cross‐sectional study (Aarsland et al.^14^) reported that p‐tau217 concentrations indicative of AD neuropathology were lower in cognitively intact individuals and in those with higher education. This suggests that cognition and education may protect against AD pathology accumulation. Longitudinal data testing this hypothesis were lacking.
2. **Interpretation**: Ten‐year longitudinal data from the population‐based Betula study (*n* = 1005) showed that plasma p‐tau217 increased with age across all participants, with the steepest increase in individuals with accelerated episodic‐memory decline. Critically, neither well‐maintained episodic memory nor higher educational attainment moderated p‐tau217 trajectories, providing no support for a protective influence on AD pathology accumulation. This null finding for education was independently replicated in the BioFINDER‐2 cohort.
3. **Future directions**: Future studies should examine whether apolipoprotein E genotype or lifestyle factors moderate p‐tau217 trajectories, incorporate neuroimaging to capture complementary pathological changes, and assess non‐linear biomarker dynamics across more than two time points.

## 2 METHODS

### 2.1 Participants

Data used in preparation of this work were obtained from the Betula Project on aging, memory, and dementia (http://www.umu.se/en/betula; Table 1), a longitudinal population‐based study conducted in Umeå, Sweden.^16^ Participants were randomly sampled from the population registry and stratified by age and sex to ensure proportional representation of the general population. Exclusion criteria included dementia at baseline, major neurological or psychiatric disorders, intellectual disability, severe sensory impairment, and non‐native Swedish language.^15^ To date, six main data collection waves (W1 to W6; 1988 to 2014) have been completed at approximately 5‐year intervals, each including a wide range of health and cognitive assessments. Blood sampling was part of the protocol at all waves, with plasma collection introduced at W3 (1998 to 2001). The present study included 1005 participants from the longitudinal Betula cohorts S1 and S3 with available plasma samples at W3. All participants were aged over 50 years and had a previously defined cognitive profile based on the intercept and slope of a five‐item episodic‐memory composite score, classifying individuals into three trajectories of episodic‐memory performance: maintainers, average performers, and decliners.^17^ Of these 1005 participants, 526 also had follow‐up plasma samples at W5 (2008 to 2010), yielding a total of 1531 samples available for plasma p‐tau217 analysis (see Supplementary Materials for a flowchart of sample selection).

**TABLE 1:** Baseline characteristics of analytical sample.

*N*	1005
**Age (SD)**	68.30 (9.91)
**Education (SD)**	9.64 (3.88)
**Female n/*N* (%)**	557/1005 (55.4%)
**APOE ε4 n/*N* (%)**	253/978 (25.9%)

### 2.2 Cognitive assessment and classification of cognitive aging profiles

Cognition was assessed using a composite measure of episodic‐memory performance. The composite included five standardized tasks: (1) immediate free recall of 16 visually and orally presented sentences, (2) delayed cued recall of nouns from the sentences, (3) immediate free recall of 16 enacted sentences, (4) delayed cued recall of nouns from enacted sentences, and (5) immediate free recall of 12 orally presented nouns. Procedures remained constant across waves. Cognitive profiles were determined following established Betula procedures and were previously described in full detail elsewhere.^17^, ^19^ At baseline, the sample includes a total of 256 maintainers, 99 decliners, and 650 average performers.

### 2.3 Educational attainment

Education was assessed as self‐reported years of formal education, recorded with half‐year precision. This variable was categorized rather than treated as continuous because the same number of years of education may correspond to different levels of relative educational attainment depending on birth cohort, reflecting historical differences in educational access. Accordingly, for analytical purposes, education was dichotomized separately within each age group (i.e., 50 to 59, 60 to 69, 70 to 79, and 80 to 99 years) based on the age‐specific median number of years of education, in order to capture relative educational attainment rather than absolute years of schooling. Participants with years of education above the age‐specific median were classified as the higher education group, whereas those with years of education equal to or below the median were classified as the lower education group. Age groups were defined based on participants’ age at p‐tau217 baseline.

### 2.4 Plasma biomarker analyses

Venous blood samples were collected at each assessment wave from non‐fasting participants during daytime hours. From W3 onward, blood collected in EDTA tubes was centrifuged to separate plasma, which was then aliquoted into secondary tubes and stored at −80°C pending biochemical analysis. A small subset of W3 samples (*N* = 94) had undergone prior thawing for earlier investigations.

Plasma p‐tau217 concentrations were quantified using a validated immunoassay platform with high analytical sensitivity and specificity for AD‐related phosphorylation at threonine 217 (Lilly p‐tau217 in‐house immunoassay).^11^, ^20^ The randomization procedure was based on the characteristics of the 1005 W3 participants. W3 samples were randomized according to age, sex, and cognitive profile to ensure an even distribution of participant characteristics across the 42 assay plates. For the longitudinal subset, the 526 W5 samples were positioned on the same plate and adjacent to the corresponding baseline (W3) sample to enhance within‐individual robustness and reduce plate‐to‐plate effects. Before finalizing the plate layout, we verified that the positioning of samples did not result in an excessively skewed distribution of dementia status or cohort affiliation (S1/S3). Plasma p‐tau217 concentrations were log‐transformed prior to statistical analyses. Importantly, because plasma was measured at W3 and W5 (10‐year follow‐up), these data do not fully overlap with the time points used to derive the cognitive aging profiles. Analyses were performed blinded to cognitive status.

### 2.5 Statistical analyses

All statistical analyses were conducted in R (version 4.4.3). To compare participants with longitudinal data to those who dropped out after W3, we used Welch's and chi‐squared tests. We then fit a linear regression model with p‐tau217 as the outcome variable and predictors including mean‐centered age, sex, and cognitive aging profile (maintainers, average performers, and decliners). A control analysis including follow‐up status (longitudinal vs drop‐out) was conducted.

Longitudinal changes in p‐tau217 were assessed using linear mixed‐effects models (lme4 package) with random intercepts for participants and fixed effects of time, mean‐centered age, sex, cognitive profile, and interactions between time × age as well as time × cognitive profile. To assess the extent to which longitudinal p‐tau217 changes were driven by individuals on a dementia trajectory, all analyses were repeated after excluding participants who developed dementia (see Ref. 16 for description of dementia assessment). Post hoc contrasts of estimated marginal means were conducted to examine changes in p‐tau217 from W3 to W5 within each cognitive profile. This was done using the emmeans package in R, with Bonferroni adjustment for multiple comparisons.

To examine whether educational attainment modified baseline levels and longitudinal changes in plasma p‐tau217, an additional mixed‐effects model included education as a predictor together with time, age, sex, and an interaction between age and education group. Statistical significance was set at *p* < 0.05 (two‐tailed) for all analyses. We applied Bonferroni correction across the six primary hypothesis‐driven tests reported in the main text: (1) two baseline cognitive profile contrasts, (2) two time × cognitive profile contrasts, (3) age × education interaction in Betula, and (4) age × education interaction in BioFINDER‐2 (corrected significance threshold α = 0.0083). Correction was also applied to estimated marginal means for p‐tau217 change within each cognitive profile.

Longitudinal trajectories of plasma p‐tau217 between W3 and W5 were visualized using individual participant line plots stratified by group. Group‐level trends were summarized by plotting mean p‐tau217 values at each time point, with shaded ribbons representing bootstrapped 95% confidence intervals. Finally, to visualize trajectories of plasma p‐tau217 in relation to educational attainment, locally estimated scatterplot smoothing curves were overlaid for each education group. Individual data points and within‐participant trajectories were retained to illustrate the distribution of observed values.

### 2.6 Replication cohort: BioFINDER‐2

We tested the potential association between p‐tau217 levels and education in a replication cohort from the Swedish BioFINDER‐2 study^11^ (https://biofinder.se/two/). This analysis included 388 cognitively unimpaired individuals aged over 50 years who had available p‐tau217 levels quantified using the same assay and education data categorized using the same approach described above for Betula. The classification of being cognitively unimpaired has been described previously.^21^

## 3 RESULTS

### 3.1 P‐tau217 in the different memory‐trajectory groups

In the full baseline sample (*N* = 1005), the mean p‐tau217 was 0.33 pg/mL (standard deviation [SD] = 0.13). A linear regression analysis adjusting for age, sex, and cognitive profile showed that p‐tau217 was higher in older age (*β* = 0.0066, standard error [SE] = 0.0010, *t* = 6.49, *p* < 0.001). There was no significant difference between males and females (*β* = −0.037, SE = 0.020, *t* = −1.85, *p* = 0.065). Compared with average performers (*M* = 0.33, SD = 0.12), decliners showed higher p‐tau217 levels (*M* = 0.37, SD = 0.15, *β* = 0.093, SE = 0.034, *t* = 2.72, *p* = 0.007) and maintainers showed lower levels (*M* = 0.32, SD = 0.13, *β* = −0.069, SE = 0.024, *t* = −2.90, *p* = 0.004). We carried out a control analysis including follow‐up status (longitudinal vs. drop‐out), with results indicating that those who remained in the study did not differ significantly at baseline from those who dropped out (*β* = −0.011, SE = 0.0218, *t* = −0.50, p = 0.618; Supplementary Materials). As a sensitivity analysis, the cross‐sectional model was repeated in the drop‐out sample only, yielding similar albeit attenuated results. Comparable findings were also observed after excluding participants with dementia, indicating that baseline differences were not primarily driven by dementia cases (Supplementary Materials).

The main analysis considered longitudinal increases in p‐tau217 in the three memory‐trajectory groups. We first carried out a linear regression analysis with age, sex, cognitive profile, and follow‐up status (drop‐out vs longitudinal), showing that p‐tau217 levels at baseline did not differ significantly between those who remained versus dropped out from the study (Table S1, *β* = −0.011, SE = 0.0218, *t* = −0.50, *p* = 0.618). A linear mixed‐effects model (Table 2) examined changes in p‐tau217 over time in the follow‐up sample. The model revealed a significant effect of time, such that p‐tau217 levels were higher at follow‐up compared with baseline across the sample (*β* = 0.179, SE = 0.016, *t*[646.8] = 11.11, *p* < 0.001). Baseline age was positively associated with p‐tau217 (*β* = 0.0066, SE = 0.0011, *t*[1243] = 6.25, *p* < 0.001), indicating that older participants exhibited higher levels. Moreover, there was a significant time × age interaction (*β* = 0.0119, SE = 0.0015, *t*[686.4] = 7.88, *p* < 0.001), suggesting that the increase in p‐tau217 from W3 to W5 was more pronounced in older participants (Figure S1). Sex was not significantly associated with p‐tau217 (*β* = −0.037, SE = 0.020, *t*[975.6] = −1.87, *p* = 0.061).

**TABLE 2:** Results of linear mixed‐effects model for longitudinal increases in plasma p‐tau217.

Predictor	Estimate	Standard error	df	*t* value	*p* value
Intercept	−1.138	0.0161	1148	−70.72	<2e‐16^***^
Time	0.179	0.0161	646.8	11.11	<2e‐16^***^
Decliners (vs Average)	0.0930	0.0355	1243	2.62	0.00894^**^
Maintainers (vs Average)	−0.0688	0.0246	1240	−2.80	0.00526^**^
Age	0.00660	0.00106	1243	6.25	5.70e‐10^***^
Sex	−0.0371	0.0198	975.6	−1.87	0.0613
Time × Decliners	0.1555	0.0433	639.2	3.59	0.000357^***^
Time × Maintainers	−0.00417	0.0289	627.6	−0.145	0.8851
Time × Age	0.0119	0.00151	686.4	7.88	1.28e‐14^***^

*Note*: The model included time, cognitive profile, age, sex, and time × cognitive profile and time × age interactions. The average group was the reference category for cognitive profile. Time compares Wave 5 with Wave 3. *N* = 1005 participants; observations = 1531.

*** *p* < 0.001.

** *p* < 0.01.

* *p* < 0.05.

Critically, the results showed an interaction between time and cognitive profile, with decliners exhibiting significantly larger increases from W3 to W5 compared with average performers (*β* = 0.155, SE = 0.043, *t*[639.2] = 3.59, *p* < 0.001; Figure 1, Figures S2 and S3). Average performers and maintainers did not differ significantly in their change over time (*β* = −0.004, SE = 0.029, *t*[627.6] = −0.15, *p* = 0.885).

**FIGURE 1:**
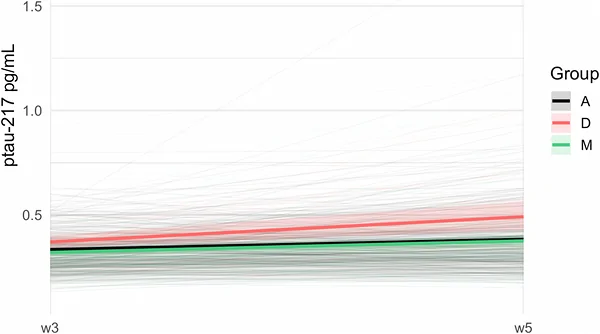
Longitudinal trajectories of plasma p‐tau217 in participants longitudinally characterized as maintainers, average performers, and decliners in episodic memory.

Sensitivity analyses indicated that the longitudinal differences between cognitive profiles were significantly attenuated after excluding participants with dementia (*N*_Maintainers_ = 30; *N*_Average_ = 135; *N*_Decliners_ = 51). After excluding dementia cases (Figure S4), decliners and average performers did not differ in p‐tau217 (*β* = 0.0387, SE = 0.0482, *t* = 0.80, *p* = 0.423) and maintainers showed only a trend (*β* = −0.0465, SE = 0.0260, *t* = −1.79, *p* = 0.074). This suggests that the stronger increase in p‐tau217 among decliners reflected emerging dementia pathology.

### 3.2 P‐tau217 in relation to educational attainment

Baseline differences in p‐tau217 between the higher and lower educational groups were small in all age cohorts (Table 3). In a linear mixed‐effects model, education group was not significantly associated with plasma p‐tau217 (*β* = 0.0302, SE = 0.0198, *t*[963.2] = 1.53, *p* = 0.1277; Table 4). The age × education group interaction was also non‐significant (*β* = 0.00176, SE = 0.00203, *t*[1035] = 0.87, *p* = 0.3859), indicating no evidence that the association between education and p‐tau217 differed by age. The longitudinal trajectories showed substantial overlap between educational groups over time (Figure 2; see Figure S5 for sex‐stratified plots). Together, these findings provide no evidence for an association between educational attainment and plasma p‐tau217.

**TABLE 3:** Educational attainment and baseline plasma p‐tau217 across age groups.

Age groups	50 to 59		60 to 69		70 to 79		80+	
***N***	176		382		261		179	
**Mean education**	12.6		10.2		8.08		7.91	
**Median education**	12		9		7		6.5	
**Median split**	High	Low	High	Low	High	Low	High	Low
**Mean education**	16.1	9.34	13.1	7.2	10.4	6.18	10.1	5.88
**Median education**	16	9	12	7	9.5	6	9	6
**Mean p‐tau217** ^*^	0.325	0.302	0.317	0.319	0.346	0.327	0.398	0.363

Abbreviation: p‐tau217, phosphorylated tau at threonine 217.

* At baseline.

**TABLE 4:** Results of linear mixed‐effects model examining associations between educational attainment and plasma p‐tau217.

Predictor	Estimate	Standard error	df	*t* value	*p* value
Intercept	−1.195	0.0323	990.5	−37.03	<2e‐16^***^
Time	0.1687	0.0132	638.7	12.77	<2e‐16^***^
Age	0.00594	0.00317	1033	1.87	0.0617
Education group	0.0302	0.0198	963.2	1.53	0.1277
Sex	−0.0357	0.0199	977.7	−1.79	0.0738
Age × Education group	0.00176	0.00203	1035	0.87	0.3859

*Note*: The model included time, age, education group, sex, and an age × education group interaction. Education group was defined using age‐specific median splits, with lower education as the reference group. Time compares Wave 5 with Wave 3. *N* = 1005 participants; observations = 1531.

Abbreviation: p‐tau217, phosphorylated tau at threonine 217.

*** *p* < 0.001.

** *p* < 0.01.

^*^ *p* < 0.05.

**FIGURE 2:**
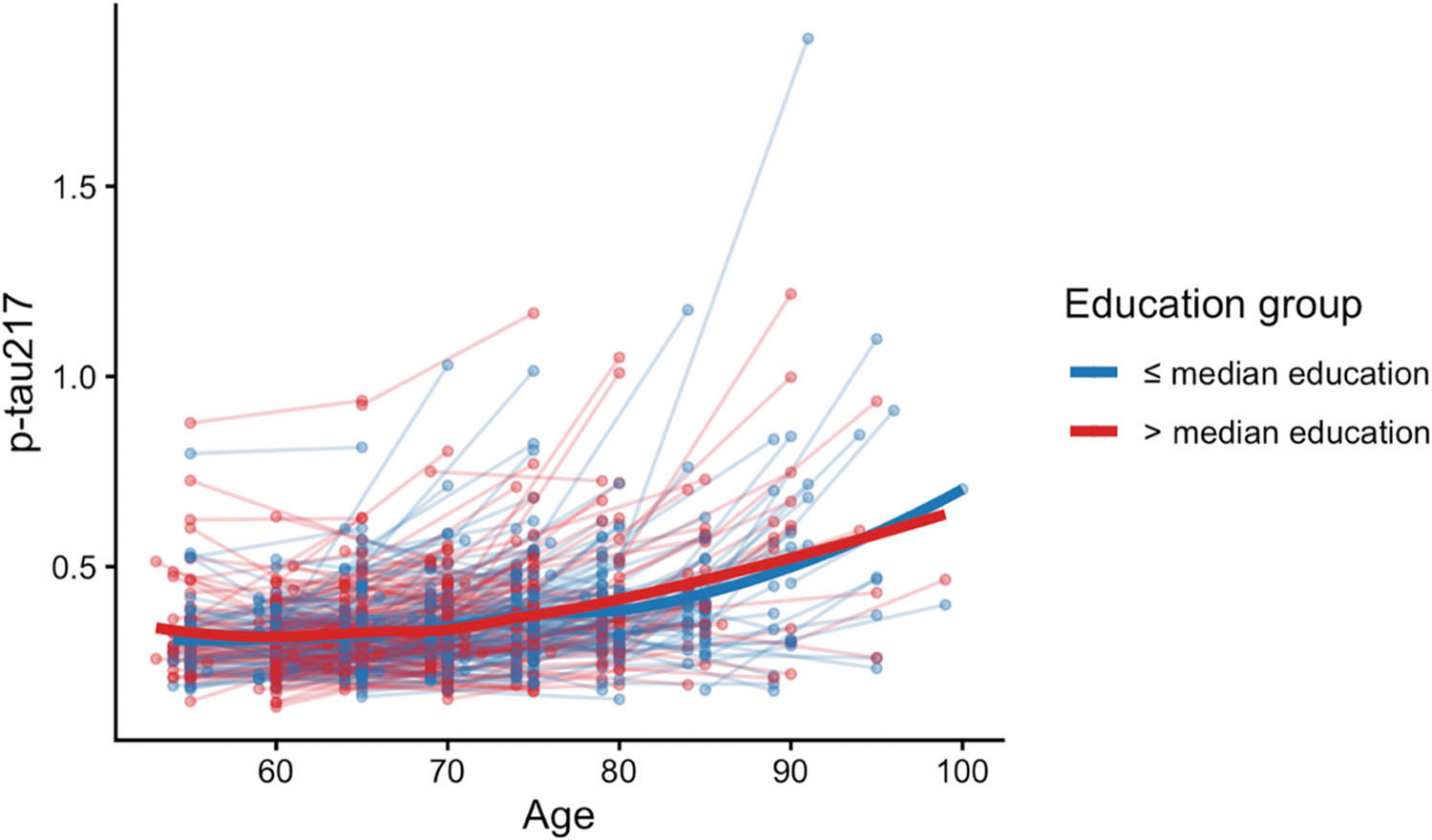
Longitudinal changes in plasma p‐tau217 as a function of age. Thin lines = individuals, bold lines = mean trajectories for higher and lower education groups.

### 3.3 Replication: educational attainment and p‐tau217 in BioFINDER‐2

In line with the main findings, analyses of data from the BioFINDER‐2 study (Table S2) revealed minor differences in p‐tau217 between individuals with higher and lower education (Table 5). A linear regression model demonstrated a positive association between age and p‐tau217 levels after controlling for sex (*β* = 0.009, SE = 0.002, *t* = 4.24, *p* < 0.001). Including an interaction term for educational level – coded as a binary variable (low vs. high) based on a median split – revealed no significant moderation effect of education on the relationship between age and p‐tau217 (age × education: *β* = −0.006, SE = 0.004, *t* = −1.492, *p* = 0.14). Additionally, sex showed no statistical association with p‐tau217 levels (*β* = −0.03, SE = 0.05, *t* = −0.718, *p* > 0.4). The distribution of p‐tau217 across age and educational groups is shown in Figure 3.

**TABLE 5:** Educational attainment and plasma p‐tau217 across age groups in the BioFINDER‐2 cohort.

Age groups	50 to 60		60 to 70		70 to 80		80+	
***N***	123		82		120		63	
**Mean education**	13.7		12.6		11.8		12.6	
**Median education**	13.0		12.0		11.0		12.5	
**Median split**	High	Low	High	Low	High	Low	High	Low
**Mean education**	16.3	11.2	14.8	10.8	14.8	9.4	16.2	9.0
**Median education**	16.0	11.0	14.0	11.0	14.0	10.0	15.0	9.0
**Mean p‐tau217**	0.162	0.147	0.180	0.190	0.203	0.202	0.197	0.240

Abbreviation: p‐tau217, phosphorylated tau at threonine 217.

**FIGURE 3:**
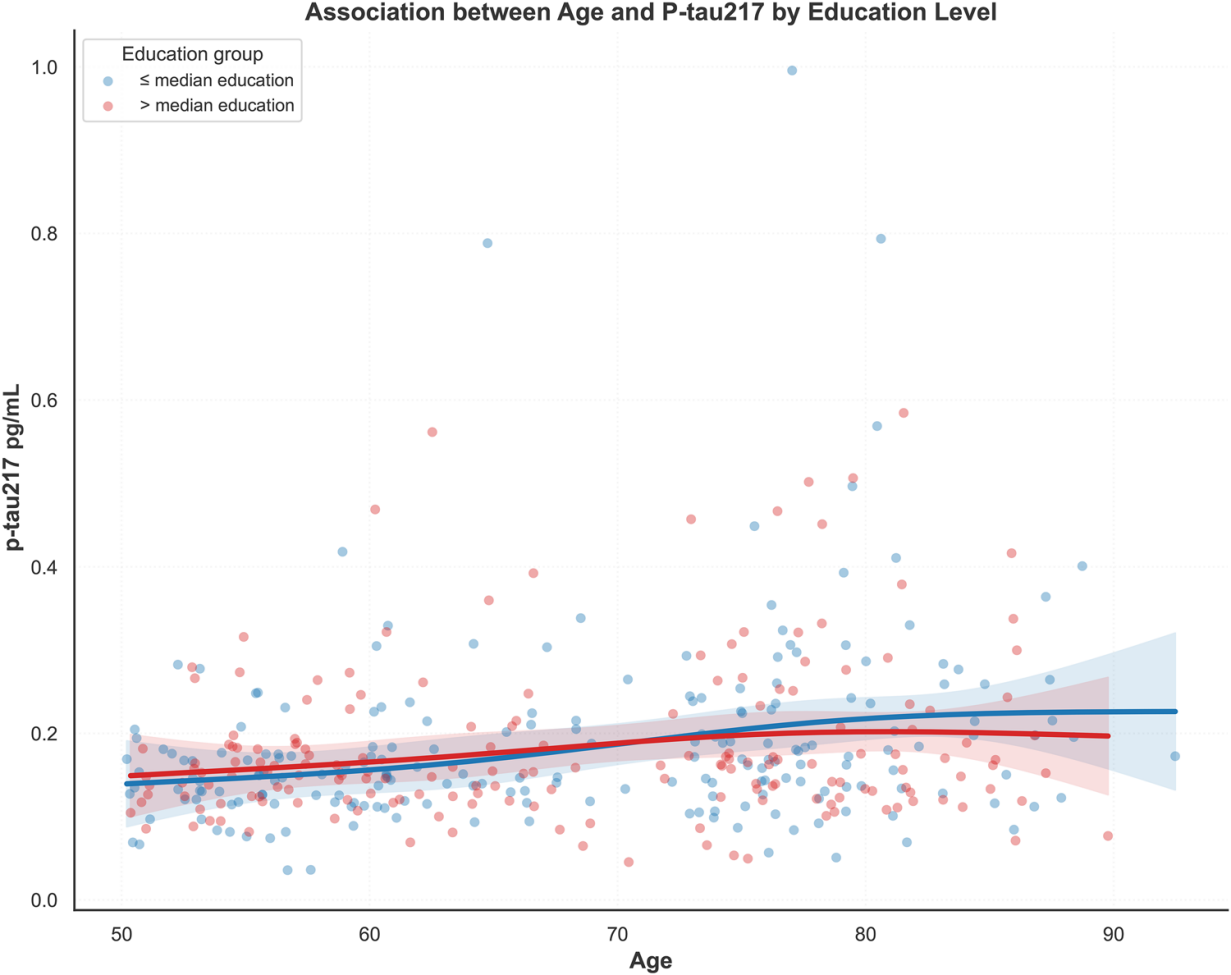
Plasma p‐tau217 as a function of age in the replication cohort. Points represent individual baseline observations, and solid lines represent the fit of a natural cubic spline stratified by educational attainment. Shaded areas depict the 95% confidence intervals.

## 4 DISCUSSION

The present study examined whether markers of cognitive resilience, namely well‐preserved cognition and higher educational attainment, are associated with lower levels or slower accumulation of plasma p‐tau217. In summary, plasma p‐tau217 increased with age over the 10‐year follow‐up period, with the steepest increase observed among individuals with accelerated episodic‐memory decline. In contrast, p‐tau217 trajectories did not differ between those with well‐maintained or age‐typical episodic‐memory change. Educational attainment was not associated with plasma p‐tau217, a null finding that was further corroborated in the BioFINDER‐2 cohort. Together, our findings do not support the notion that higher education or well‐maintained memory protects against AD neuropathological changes.

We found that p‐tau217 increased significantly over time in all memory‐trajectory groups and that the increase was influenced by baseline age with a steeper increase for older participants. The p‐tau217 increase was significantly greater for memory decliners. However, after excluding incident dementia cases, this longitudinal difference was markedly attenuated, suggesting that the accelerated p‐tau217 increase among memory decliners was driven by pathology. These observations are consistent with past findings of a higher p‐tau217 burden in older individuals with MCI and dementia.^7^, ^14^, ^22^, ^23^, ^24^ Differences between memory maintainers and average performers have been demonstrated for genetic, epigenetic, and lifestyle factors.^17^, ^25^ Here we found that longitudinal p‐tau217 trajectories overlapped for these groups, which is in line with findings of no cross‐sectional differences in p‐tau181 burden between Superagers and age‐typical controls.^26^

For educational attainment, we found highly similar increases in plasma p‐tau217 levels over the 10‐year follow‐up for individuals with higher or lower education. These results were replicated in an independent BioFINDER‐2 cohort of cognitively unimpaired individuals, in which no association was also observed. As noted in the introduction, Aarsland et al.^14^ did find that individuals with higher education had a lower prevalence of plasma p‐tau217 verified AD, in particular in the oldest age groups. They noted that their findings supported a protective influence of education, for instance, by contributing to a *cognitive reserve*.^27^, ^28^ In contrast, our results that education does not protect against p‐tau217 buildup is consistent with prior evidence showing that education does not influence the rate of age‐related cognitive decline and only has modest influences on initial neurocognitive levels.^29^, ^30^, ^31^, ^32^ Together, these suggest that higher education supports initial brain and cognitive development but may not protect against detrimental age‐related brain changes.

Several factors may help explain the discrepant findings between the present study and the work by Aarsland et al.^14^ First, their analyses focused on cross‐sectional prevalence of biomarker positivity using predefined plasma p‐tau217 cut‐offs to classify individuals with AD. Our analyses examined continuous biomarker levels and within‐person longitudinal change. Second, differences in the age composition of the samples may contribute to divergent results. In Aarsland et al.,^14^ the analyses of education in relation to AD were conducted in participants aged 70 years and older. In contrast, our sample included participants from the early 50s onward. Education‐related differences may therefore be more difficult to detect in younger groups where AD pathology is less common and only become apparent at older ages when pathological burden increases. The education classification also differed across studies, which further limits direct comparisons. Moreover, neither study fully accounted for potential confounding influences such as comorbid medical conditions, which may vary between populations and influence p‐tau217 levels or their relationship with education. Lastly, HUNT included participants across the dementia spectrum and had a substantially larger sample size, whereas Betula excluded dementia at baseline and has an extensive longitudinal follow‐up.

The Betula Project provides a unique combination of population‐based data, comprehensive cognitive assessments, and repeated blood sampling across multiple waves, enabling the examination of longitudinal cognitive and biomarker trajectories.^16^ The population‐based design of Betula is an important strength and supports the generalizability of the findings beyond clinic‐based cohorts. Nevertheless, replication in more diverse populations is necessary. Our study also comes with certain limitations. Biomarker analyses were limited to two time points approximately a decade apart, restricting assessment of non‐linear changes. Although the interval between assessments allowed us to examine within‐person change over a substantial time period, two time points still provide less precise and sensitive estimates of longitudinal change compared with designs with more repeated measurements. This also limits the ability to estimate individual‐specific slopes. In addition, cognitive change and p‐tau217 change were not modeled over fully concurrent intervals. Future longitudinal work should directly test change‐on‐change associations when possible and combine plasma p‐tau217 with neuroimaging measures such as hippocampal atrophy and indices of vascular–metabolic burden. We did not have access to health‐related variables that may influence plasma p‐tau217 concentrations, including kidney function and anemia status. These should be considered in future studies with available clinical data. Lastly, examining how p‐tau217 interacts with APOE genotype and lifestyle factors will be important.

In summary, our findings underscore the value of plasma p‐tau217 for detecting early pathological changes in population‐based settings but provide no support that individuals with well‐maintained episodic memory or high educational attainment are protected against p‐tau217 accumulation.

## CONFLICT OF INTEREST STATEMENT

OH is an employee of Eli Lilly and Lund University. SP has acquired research support (for the institution) from Avid Radiopharmaceuticals and ki elements through Alzheimer's Drug Discovery Foundation. In the past 3 years, he has received consultancy/speaker fees from BioArtic, Danaher, Eisai, Eli Lilly, Novo Nordisk, Roche, and Sanofi. All other authors declare no conflicts of interest. Author disclosures are available in the Supporting Information.

## CONSENT STATEMENT

All participants or their representatives provided written informed consent.

## Supporting information



Supporting Information

Supporting Information

## Data Availability

Due to legislation and ethical restrictions, the data are not publicly available. Access may be granted upon reasonable request to the corresponding author, approval by the Betula Steering Group (umu.se/en/betula), and a confidentiality assessment.
