# Supplementary material for: Stable episodic memory and higher education do not influence the rate of Alzheimer's disease pathology as measured by plasma p‐tau217
Source: Alzheimers Dement. 2026 Nov 4;22(11):e71920. doi: 10.1002/alz.71920 (PMC13736553; doi:10.1002/alz.71920)
Supplement: Supplementary file 2 [file ALZ-22-e71920-s001.docx]

**Supplementary Materials**

**Flowchart of study sample selection from the Betula project**

**Supplementary Table 1**:

Betula Longitudinal and Drop-out samples

| **Sample** | **Memory Profile** | | |
| --- | --- | --- | --- |
| *Longitudinal*  N  P-tau217 (pg/ml)  Age (years)  Sex (f/m) | *Maintainer*  142  0.30 (0.10)  67.00 (8.75)  99/43 | *Average*  335  0.32 (0.10)  63.92 (8.03)  177/158 | *Decliner*  49  0.35 (0.12)  62.92 (6.08)  25/24 |
| *Drop-out*  N  P-tau217 (pg/ml)  Age (years)  Sex (f/m) | *Maintainer*  114  0.34 (0.16)  74.48 (9.68)  68/46 | *Average*  315  0.34 (0.14)  71.50 (10.15)  159/156 | *Decliner*  50  0.39  72.42 (10.18)  29/21 |

*Note: M (SD) for P-tau217 and Age; Number for Sex*

**Supplementary Table 2.**

Characteristics of the BioFINDER-2 cohort.

| **Variable** | **BioFINDER-2 (N=388)** |
| --- | --- |
| Age, years | 68 (11) |
| Age, range | 50 - 92 |
| Sex, n. females (%)  Education, years | 219 (56%)  13 (4) |
| p-tau217 levels (pg/ml, std.) | 0.186 (0.108) |

**Supplementary Figure 1.** Individual trajectories for p-tau217 changes. The bold orange line indicates mean change, estimated using a Generalized Additive Mixed Model.

**Supplementary Figure 2.** Baseline and follow-up differences in p-tau217 among maintainers, average performers, and decliners.

**Supplementary Figure 3.** Trajectories of p-tau217 for Average, Decliners, and Maintainers.

**Supplementary Figure 4.** Longitudinal trajectories of p-tau217 after excluding individuals with dementia

**Supplementary Figure 5.** Individual and mean trajectories of plasma p-tau217 by sex.
